# Nonalcoholic Fatty Liver Disease: Different Classifications Concordance and Relationship between Degrees of Morphological Features and Spectrum of the Disease

**DOI:** 10.1155/2014/526979

**Published:** 2014-12-02

**Authors:** Juliana Maya Monteiro, Geysa Maya Monteiro, Adriana Caroli-Bottino, Vera Lucia Pannain

**Affiliations:** ^1^Postgraduate Program, Department of Pathology, University Hospital, Faculty of Medicine, Federal University of Rio de Janeiro, Avenida Prof. Rodolpho Paulo Rocco 255, Cidade Universitária, 21941-913 Rio de Janeiro, RJ, Brazil; ^2^Faculty of Medicine, Severino Sombra University, Avenida Expedicionário Oswaldo de Almeida Ramos 280, Centro, 27700-000 Vassouras, RJ, Brazil; ^3^Department of Pathology, University Hospital, Faculty of Medicine, Federal University of Rio de Janeiro, Avenida Prof. Rodolpho Paulo Rocco 255, Cidade Universitária, 21941-913 Rio de Janeiro, RJ, Brazil

## Abstract

The morphological features of nonalcoholic fatty liver disease (NAFLD) range from steatosis to nonalcoholic steatohepatitis (NASH) and cirrhosis. Liver biopsy remains the main tool for NASH diagnosis and many histological systems to diagnose and grade NAFLD were proposed. We evaluated the relationship among NAFLD activity score (NAS), histological diagnoses (non-NASH, possible NASH, and definite NASH), and histological algorithm proposed by Bedossa et al.; additionally the degrees of morphological features were semiquantified and correlated with non-NASH and NASH. Seventy-one liver biopsies were studied. The agreement among the three systems considering NASH and non-NASH was excellent (Κ = 0.96). Among the 22 biopsies with NAS 3-4, 72.7% showed to be NASH according to Bedossa's algorithm. The degree of steatosis, ballooning, lobular inflammation, and fibrosis stage were correlated with NASH (*P* < 0.001). Fibrosis stage 1 was also found in non-NASH. Over the spectrum of NAFLD, no association was observed between intensity of steatosis and fibrosis grade. The degrees of lobular inflammation showed association with fibrosis stage (*P* < 0.0001). In conclusion, there is agreement among different NAFLD classifications and NAS > 4 may be a better cutoff from which to consider NASH diagnosis; besides the highest degrees of steatosis, ballooning, inflammation, and fibrosis are associated with NASH.

## 1. Introduction

Nonalcoholic fatty liver disease (NAFLD) is a clinicopathological entity that could be the main cause of chronic liver disease in the coming decades [1]. It is closely associated with states of insulin resistance such as obesity, hyperlipidemia, and type II diabetes. The morphological features of NAFLD range from simple steatosis, which frequently has a benign course, to nonalcoholic steatohepatitis (NASH) with or without fibrosis that may progress to cirrhosis [2]. Although some noninvasive biomarkers have been developed to establish diagnosis and evaluate fibrosis [3–5], liver biopsy remains the main tool for confirming the NASH diagnosis and also to provide information about its prognosis.

In 1980, Ludwig et al. were the first to recognize nonalcoholic steatohepatitis as a histological entity, very similar to the one already known in the liver from patients with alcohol abuse [6]. Almost two decades later, Matteoni et al. proposed a histological system for NAFLD that classified the biopsies into four subgroups based on the following features: steatosis, necroinflammatory lesions, ballooning, Mallory-Denk hyaline, and fibrosis [7]. Types 1 and 2 were histological forms of non-NASH, while biopsies of types 3 and 4 were histologically and clinically similar to NASH. The patients with type 1 disease (steatosis alone) showed the best outcome when compared to types 3 and 4 (steatosis, ballooning degeneration, and Mallory-Denk hyaline or fibrosis), in which cirrhosis and liver-related deaths were more frequent.

In the same year, Brunt et al. proposed a grading and staging system for NASH [8]. The grading was based on a combination of steatosis, ballooning, and portal and lobular inflammation, while the staging took account mainly of the fibrosis localization, whether perisinusoidal/pericellular, portal, bridging, or cirrhosis. In 2005, the Nonalcoholic Steatohepatitis Clinical Research Network (NASH-CRN) Pathology Committee proposed the NAFLD activity score (NAS), which is the sum of each histological component semiquantitatively evaluated as follows: steatosis (0–3), ballooning (0–2), and lobular inflammation (0–3). Cases with NAS 0–2 were not considered steatohepatitis, 3-4 possible steatohepatitis, and ≥5 definite steatohepatitis [9]. The authors emphasized that NAS should not replace the histological diagnosis, as it was proposed for following treatment and disease progression [9]. Several years later, the same group observed in a large cohort that the diagnosis of NASH was not always correlated with NAS values [10]. Recently, Bedossa et al. [11] proposed a NASH histological algorithm based on conclusions of the American Association for the Study of Liver Disease (AASLD). However, despite some previous studies on the subject, the NAFLD score is still controversial [12], and more histological studies to investigate its applicability in other centers are needed.

The aim of this study was to evaluate the relationships among NAFLD score, histological diagnosis (non-NASH, possible NASH, and definite NASH), and the histological algorithm proposed by Bedossa et al. [11]; additionally the degrees of morphological characteristics were correlated with non-NASH and NASH samples.

## 2. Material and Methods

Seventy-one patients with histological diagnosis of NALFD on liver biopsy were enrolled in this study. Patients with clinical and laboratory evidence of other liver diseases and/or daily alcohol ingestion ≥20 g were excluded. The institutional ethics committee approved this study.

### 2.1. Histological Analyses

The liver sections (5 *μ*m thickness) were stained with hematoxylin-eosin, Masson's trichrome, and Picrosirius red. Each liver biopsy was assessed independently by two liver pathologists (VP and AC) and diagnosed as non-NASH, possible NASH, or definite NASH using a pattern of recognition (histological diagnosis) [13], without knowledge of the previous diagnosis. In the event of different diagnoses between the pathologists, consensus was achieved between them. The morphological features of steatosis (grade I: >5–33%, grade II: >33–66%, and grade III: >66%), ballooning (0–2), lobular inflammation (0–3), and fibrosis stage (1: perisinusoidal or periportal; 2: perisinusoidal and periportal; 3: bridging fibrosis; 4: cirrhosis) were semiquantitatively evaluated according to NASH CRN criteria [9]. Subsequently, the NAS was applied (<3, non-NASH; 3-4, possible NASH; >5, NASH) [9]. Bedossa's histological algorithm was also used to categorize the same biopsy slides as NAFLD (≥5% of hepatocytes with steatosis) and NASH (the same steatosis cutoff plus any degree of hepatocellular ballooning and lobular inflammation) [11].

### 2.2. Statistical Analyses

Concordance of kappa (*Κ*) coefficient [12] was used to assess the agreement among NAFLD score, histological diagnosis, and Bedossa's histological algorithm. The association between variables degrees (steatosis, ballooning degeneration, inflammatory infiltration, and fibrosis) in non-NASH and NASH was verified by statistical independence test (Wilks G2). *P* values < 0.05 were considered significant.

## 3. Results

All patients demonstrated elevated serum levels of aminotransferases and steatosis on ultrasound. Some of them had diabetes mellitus and/or were overweight. Most patients were women (69%), and the overall mean age was 55.6 years at the time of biopsy.

The histological diagnosis according to the pattern of recognition was NASH in 50.8% of the biopsies, non-NASH in 23.9%, and possible NASH in 25.3%. Using NAS scoring, 30.9% of patients were each classified as NAS < 3 and NAS 3-4, respectively, and 38.2% were NAS ≥ 5. According to Bedossa's histological algorithm, 61.9% of the biopsies demonstrated NASH, and 38.1% demonstrated NAFLD (non-NASH). The agreement among these three forms of evaluation with regard to NASH and non-NASH was excellent (*Κ* = 0.96). Comparing histological diagnosis and NAS, which both include possible NASH, the agreement was good (*Κ* = 0.69) for NASH, excellent (*Κ* = 0.82) for non-NASH, and moderate (*Κ* = 0.51) for possible NASH.

We then verified in what pathway of Bedossa's histological algorithm the 22 biopsies with NAS 3-4 were classified. We found that 72.7% of these biopsies met at least the minimum criteria for NASH (steatosis grade I, lobular inflammation < 2, and rare ballooning). By contrast, all six biopsies diagnosed as non-NASH had steatosis grade II, and 50% showed ballooning. Furthermore, both lobular inflammation and ballooning were observed in the same percentage (86.3%) of biopsies with NAS 3-4. Also, 85.7% of NAS = 4 biopsies were diagnosed as NASH.

### 3.1. Degree of Morphological Features and Fibrosis Grade in NASH and Non-NASH Biopsies according to Bedossa's Histological Algorithm [11]

The higher degree of steatosis was observed exclusively in NASH. Grade 2 steatosis was also predominant in NASH, as grade 1 was in non-NASH (Table 1). NASH was positively correlated with the level of steatotic hepatocytes (*P* < 0.001). Frequent hepatocytes with ballooning degeneration were found in 34.09% of NASH patients, while few such hepatocytes were observed in the remaining 65.91% of NASH patients. Although also present in non-NASH, ballooning hepatocytes were few (44.44%). The degree of ballooning was associated with NASH diagnosis (*P* < 0.001). Slight lobular inflammation (<2 foci) predominated in NASH (63.63%) and non-NASH (22.22%) biopsies. In the remaining cases of NASH (36.37%) the infiltrate was moderate (2–4 foci), whereas infiltrate was absent in 77.78% of non-NASH cases. Severe infiltrate (>4 foci) was not found on any biopsies diagnosed as NAFLD. Like steatosis and ballooning injury, lobular inflammation was strongly correlated with a diagnosis of NASH (*P* < 0.001) (Table 1).

Fibrosis was present in 67.61% of all patients, corresponding to 75% of NASH patients. Of those, 27.3% each had stages 1 and 2 fibrosis, 13.6% had bridging fibrosis (stage 3), and 6.8% had cirrhosis (stage 4). Fibrosis stage was positively associated with NASH diagnosis (*P* < 0.001). Notably, 55.5% of non-NASH patients had fibrosis, but it was mainly stage 1 (93.3%) and was located in perisinusoidal zone 3. Bridging fibrosis and cirrhosis were not observed in non-NASH biopsies (Table 1).

### 3.2. Association between Morphological Features and Fibrosis Grade in NASH and Non-NASH Biopsies according to Bedossa's Histological Algorithm [11]

With regard to the possible association between fibrosis and degree of steatosis in NASH, we observed that fibrosis stages 1 and 3 were predominant in patients with steatosis grade 1 (each comprising 25% of cases), followed by fibrosis stage 2 (16.67%). Patients with moderate steatosis were similar, with the same percentage of biopsies (28.57%) each classified as stages 1 and 2, while 9.52% each were stages 3 and 4. Furthermore, in non-NASH patients, fibrosis stage 1 was seen in 51.85% of cases with steatosis. Over the spectrum of NAFLD from mild steatosis to NASH, no association was observed between intensity of steatosis and fibrosis grade (*P* = 0.774) (Tables 2 and 3).

No significant difference was detected between fibrosis grade and extent of ballooning in either NASH (*P* = 0.252) or non-NASH (*P* = 0.726). In NASH, fibrosis was observed in 75.86% of the biopsies with little ballooning and in 73.33% biopsies with frequent ballooning, whilst the non-NASH patients with fibrosis grade 1 were almost equally distributed between those with and without ballooning (Tables 2 and 3).

In NASH, among the cases with slight lobular inflammation, 75.86% showed some fibrosis, 50% with stages 1 and 2. All cases with moderate inflammation had fibrosis, 62.65% at stages 1 and 2 and 37.5% at stages 3 and 4. Of nine biopsies with fibrosis stages 3 and 4, 66.67% showed moderate inflammation, while in 35 patients without fibrosis or with fibrosis stages 1 and 2, only 28.57% had such a degree of inflammation. This association was significant (*P* < 0.0001) (Table 2). Among non-NASH patients, even those without inflammation, 57.14% had fibrosis stage 1. In six patients with mild inflammatory infiltrate, two demonstrated fibrosis stage 1 and one demonstrated stage 2, with rates of 33.33% and 16.67%, respectively (Table 3).

## 4. Discussion

The present study demonstrated that the agreement among different diagnostic approaches (histological diagnosis, NAS, and Bedossa's algorithm) was excellent for NASH and non-NASH diagnosis. Between the classifications that include possible NASH (histological diagnosis and NAS), agreement was good for NASH, moderate for possible NASH, and excellent for non-NASH. Even across these two scenarios, classifications with and without a designation of possible NASH, there was agreement among different sets of diagnoses, although we found better agreement among these classifications than other authors [10, 11]. This difference may be due to the sample size of our study or because all diagnoses were reached in consensus between pathologists using strict pathological criteria [14] for NASH, non-NASH, and possible NASH. However, this issue is still under discussion in the literature [14, 15]. As mentioned before, the NAS scoring system should not be used to establish NASH diagnosis [9]. This recommendation was confirmed by the same research group in 2011 in a large series of adult patients enrolled in the NASH Clinical Research Network [10]. They found that, in patients with biopsies of NAS ≥ 5.86% had steatohepatitis and 3% had no steatohepatitis; for NAS ≤ 4, 29% had steatohepatitis and 42% had no steatohepatitis [10].

The histological diagnoses applied in the present study and in Bedossa's algorithm [11] used the same minimal criteria for steatohepatitis (>5% steatosis, any ballooning, and inflammation) [14]. However, the previous study considered only two diagnoses (steatohepatitis and not steatohepatitis); borderline steatohepatitis was not included. In clinical practice, the pathological diagnosis of steatohepatitis can be difficult to characterize, leading to the recommendation for possible NASH when morphological features are insufficient for a definitive NASH diagnosis [14, 16].

Consistent with a previous study [16], we diagnosed 30.9% of biopsies as possible steatohepatitis. Those cases were more often associated with definitive steatohepatitis (72.7%) according to Bedossa's algorithm [11], demonstrating that, in cases of NAS 3-4, the histology is not benign. Previous studies have linked NAS values <3 and >4 to the absence and presence of steatohepatitis, respectively [9, 11]. However, they did not find any correlation of NAS values 3 and 4 with non-NASH or NASH. The authors believe that inclusion of steatosis in NAS is the reason for the discrepancy between NAS and NASH diagnosis [11]. In our study, 87.5% of biopsies classified as NAS 4 were diagnosed with NASH, while 22.5% were non-NASH, demonstrating that if NAS ≥ 5 is used as the threshold for NASH diagnosis, many NASH cases with scores below 5 may be missed. It is possible that NAS > 4 is a better cutoff from which to consider NASH diagnosis. Hjelkrem et al. also observed that NAS ≥ 4 has high sensitivity and specificity for NASH diagnosis [17]. Other authors observed steatohepatitis in 29% of patients with NAS ≤ 4, although notably this analysis included cases with a score below 4 [10]. Excluding scores below 4 would have increased the rate of steatohepatitis.

In the present study steatohepatitis was significantly related with the degree of steatosis, ballooning, and lobular inflammation. Steatosis > 5% is a* sine qua non* condition for NAFLD diagnosis, regardless of the classification used. Patients may have a significant degree of steatosis without steatohepatitis, but we did not observe severe steatosis in any cases of nonsteatohepatitis. Nonetheless, biopsies with severe and moderate steatosis were more likely to be diagnosed as definite steatohepatitis, whilst slight steatosis predominated in non-NASH. Chalasani et al. also observed a relationship between histological grade of steatosis and steatohepatitis [18].

Ballooning hepatocytes are considered a significant histological feature to the determination of steatohepatitis [10]. However, recognizing ballooning hepatocytes is sometimes an issue, because biopsies do not always meet the classical definition of ballooning. Indeed, this difficulty is also reflected in decreased agreement among pathologists (*Κ* values) [10, 11, 19] and remains a concern to steatohepatitis diagnosis. We found ballooning hepatocytes in 86.36% of possible steatohepatitis cases based on histological diagnosis, many of which were graduated as few cells. This finding reinforces the importance of ballooning degeneration for steatohepatitis [10], since this feature is not always precisely identified. Immunostaining for antibodies against keratins 8 and 18 can identify such cells with more confidence, because they are reduced in ballooning hepatocytes [20].

Lobular inflammation is one of the basic features required for a diagnosis of steatohepatitis. In this study, lobular inflammatory infiltrate was correlated with the diagnosis of definite steatohepatitis, as observed in other series [9]. Typically inflammation is slight and consists of small foci of lymphocytes and macrophages, sometimes associated with polymorphs [2]. We also observed only slight or moderate inflammation in all cases.

Fibrosis may be present in patients with NAFLD and therefore has been considered a diagnostic criterion [7]. In our study, the stage of fibrosis was statistically significantly related with NASH, as previously described by others [9]. In spite of the lack of this association in non-NASH, fibrosis was present in 55% of patients, particularly perisinusoidal in zone 3. A recent study in non-NASH patients with follow-up biopsies demonstrated that those with mild inflammation and fibrosis, though minimal, had a greater risk of disease progression compared to patients with steatosis alone [21]. Fibrosis stage was associated with the degree of inflammation in NASH, but not with the amount of ballooning or steatosis, in the present study.

Gramlich et al. concluded that hepatocyte injuries (ballooning and Mallory-Denk hyaline) were the most prominent histological features associated with hepatic fibrosis in NAFLD [22]. Inflammation and fibrogenesis are thought to be closely related in the pathogenesis of NAFLD. Patients with risk factors for NAFLD, especially insulin resistance, have an increased influx of fatty acids into hepatocytes. These substances are toxic to cells, leading to oxidative stress and inflammatory stimuli, including Kupffer cell and platelet activation, monocyte infiltration, and release of cytokines responsible for activation of stellate cells and fibrogenic processes [23]. It remains unclear why some patients had more or less inflammation, but certain risk factors are known to be linked to fibrosis progression, including obesity [7], female sex, age greater than 60 years, and type II diabetes mellitus, a condition also found in the majority of patients in our study [24].

In summary, this study shows that there is agreement among different NAFLD classifications and NAS > 4 may be a better cutoff from which to consider NASH diagnosis; besides highest degrees of steatosis, cell injury, inflammation, and fibrosis are associated with NASH, although fibrosis is also observed in half of the patients with non-NASH disease.

## Figures and Tables

**Table 1:** Degree of morphological features and fibrosis stage in NASH and non-NASH biopsies according to Bedossa's histological algorithm.

Morphological features	NAFDL		*P* value
	NASH	Non-NASH	
Steatosis			*P* < 0.001
5–33%	12 (27.27%)	22 (81.48%)	
33–66%	21 (47.73%)	5 (18.52%)	
>66%	11 (25%)	0	
Ballooning			*P* < 0.001
Absent	0	14 (51.85%)	
Few	29 (65.91%)	12 (44.44%)	
Frequent	15 (34.09%)	1 (3.71%)	
Lobular inflammation			*P* < 0.001
Absent	0	21 (77.78%)	
<2 foci/field	28 (63.63%)	6 (22.22%)	
2–4 foci/field	16 (36.37%)	0	
Fibrosis			*P* < 0.001
Absent	11 (25%)	12 (44.5%)	
Perisinusoidal or periportal	12 (27.3%)	14 (51.8%)	
Perisinusoidal and periportal	12 (27.3%)	1 (3.7%)	
Bridging fibrosis	6 (13.6%)	0	
Cirrhosis	3 (6.8%)	0	

Total	44 (61.98%)	27 (38.02%)	

NAFLD: nonalcoholic fatty liver disease; NASH: nonalcoholic steatohepatitis.

**Table 2:** Association between morphological features and fibrosis grade in NASH biopsies according to Bedossa's histological algorithm.

Morphological features	Fibrosis					*P* value
	Absent	Stage 1	Stage 2	Stage 3	Stage 4	
Steatosis degrees						*P* = 0.782
1	4 (33.33%)	3 (25%)	2 (16.67%)	3 (25%)	0	
2	5 (23.82%)	6 (28.57%)	6 (28.57%)	2 (9.52%)	2 (9.52%)	
3	2 (18.19%)	3 (27.27%)	4 (6.36%)	1 (9.09%)	1 (9.09%)	
Ballooning						*P* = 0.252
Few	7 (24.14%)	10 (34.48%)	6 (20.69%)	5 (17.24%)	1 (3.45%)	
Frequent	4 (26.67%)	2 (13.33%)	6 (40%)	1 (6.67%)	2 (13.33%)	
Lobular inflammation						*P* < 0.001
<2 foci/field	11 (39.29%)	8 (28.57%)	6 (21.43%)	2 (7.14%)	1 (3.57%)	
2–4 foci/field	0	4 (25%)	6 (37%)	4 (25%)	2 (12.5%)	

**Table 3:** Association between morphological features and fibrosis grade in non-NASH biopsies according to Bedossa's histological algorithm.

Morphological features	Fibrosis			*P* value
	Absent	Stage 1	Stage 2	
Steatosis degree				*P* = 0.774
1	10 (45.45%)	11 (50%)	1 (4.55%)	
2	2 (40%)	3 (60%)	0	
3	0	0	0	
Ballooning				*P* = 0.726
Absent	7 (50%)	6 (42.86%)	1 (7.14%)	
Few	5 (41.67%)	7 (58.33%)	0	
Frequent	0	1 (100%)	0	
Lobular inflammation				*P* = 0.163
Absent	9 (42.86%)	12 (57.14%)	0	
<2 foci/field	3 (50%)	2 (33.33%)	1 (16.67%)	
2–4 foci/field	0	0	0	
